# Supplementary material for: Genetic Diversity in Diospyros Germplasm in the Western Caucasus Based on SSR and ISSR Polymorphism
Source: Biology (Basel). 2021 Apr 19;10(4):341. doi: 10.3390/biology10040341 (PMC8073590; doi:10.3390/biology10040341)
Supplement: Supplementary file 1 [file biology-10-00341-s001.zip › Suppl Fig 1.pdf]

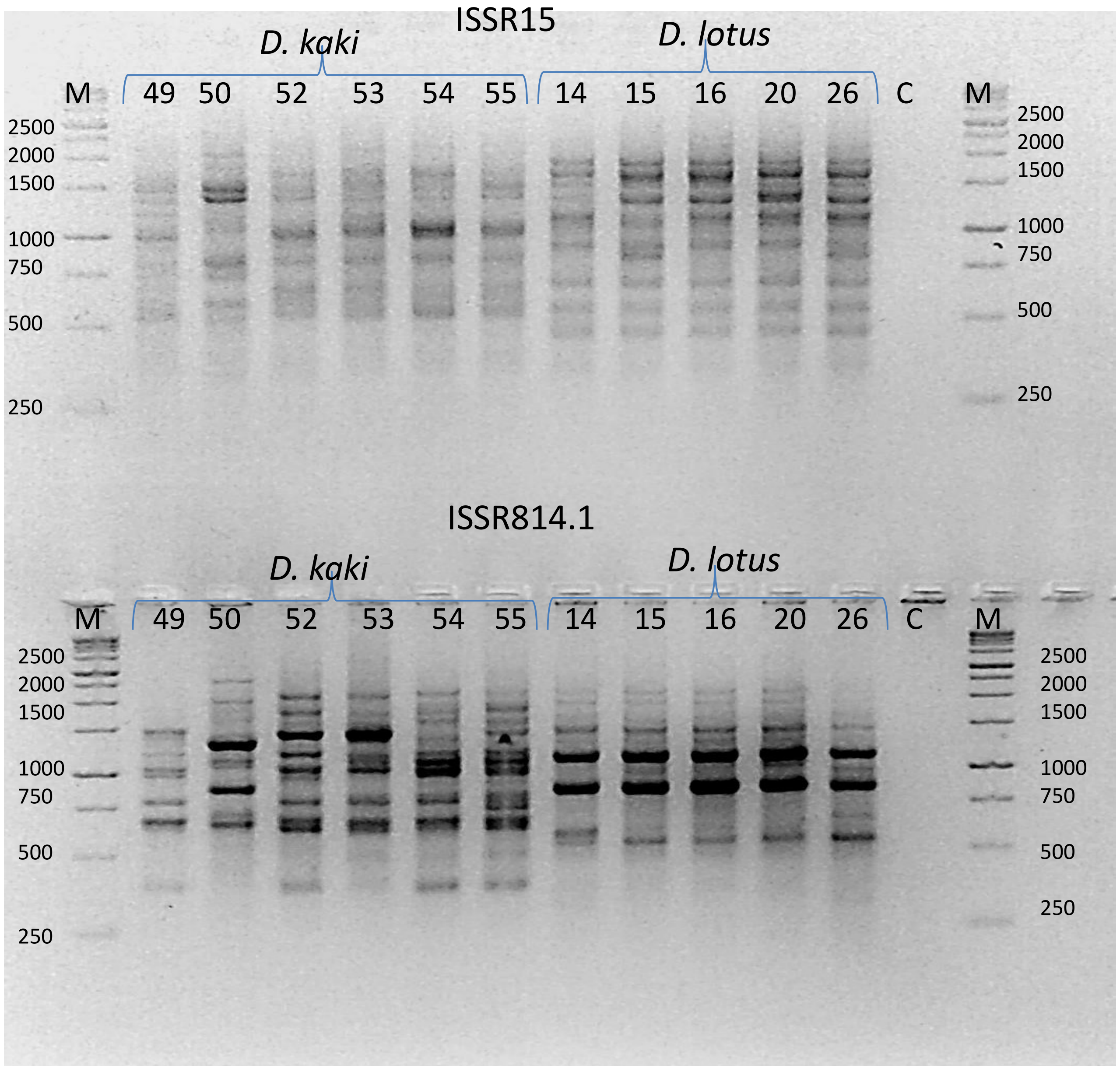

M – size marker, C – control; 49- D.kaki cv. ‘Tamopan’; 50- D.kaki cv. ‘Meader’; 52- D.kaki cv. ’Hachiya’, 53- D.kaki cv. ’Fuyu’, 54- D.kaki cv. ’Hyakume’, 55- D.kaki cv. ’Zenjimaru’; 14, 15,16,20,26 – D.lotus accessions.

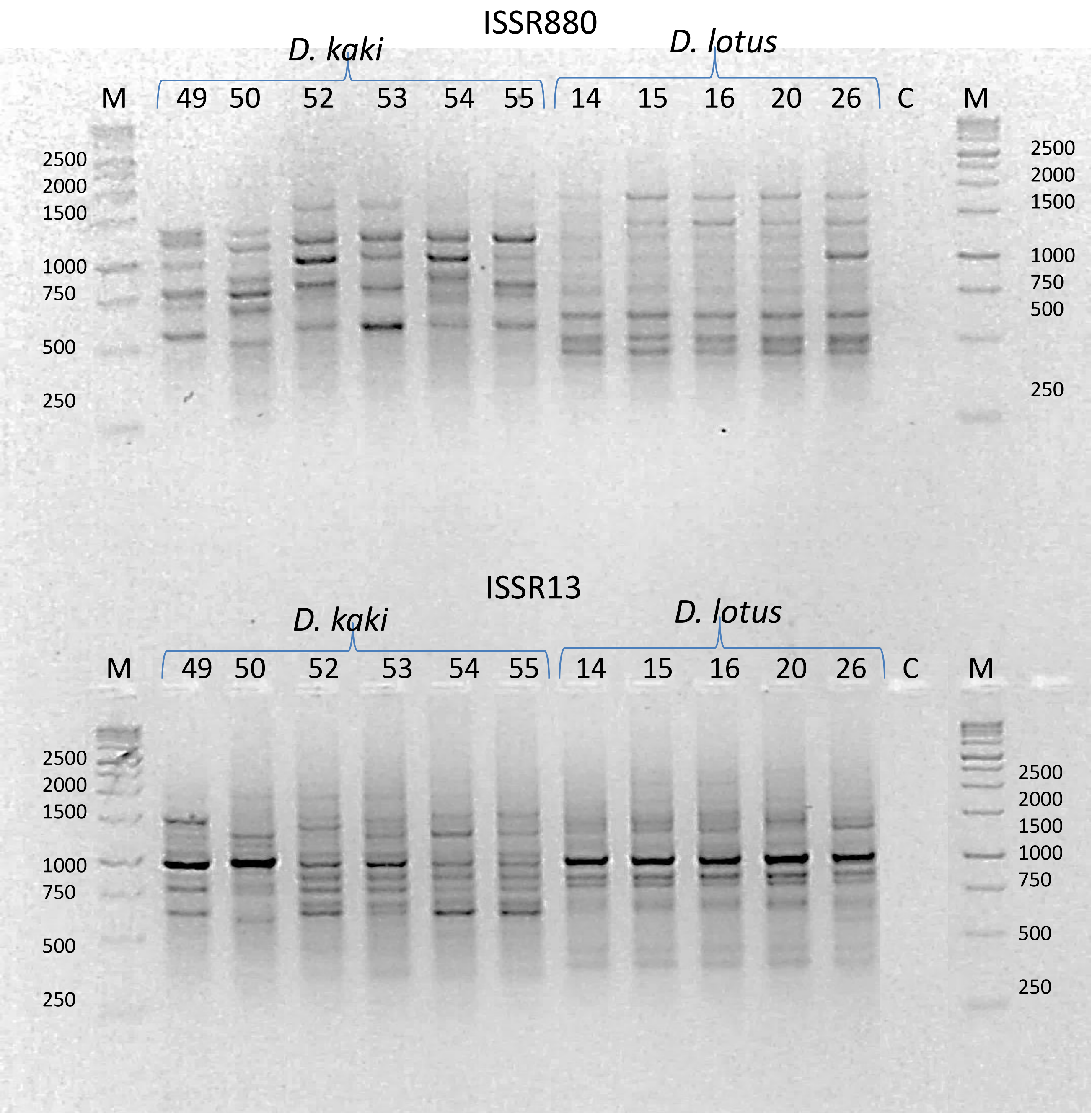

M – size marker, C – control; 49- D.kaki cv. 'Tamopan'; 50- D.kaki cv. 'Meader'; 52- D.kaki cv. 'Hachiya', 53- D.kaki cv. 'Fuyu', 54- D.kaki cv. 'Hyakume', 55- D.kaki cv. 'Zenjimaru'; 14, 15,16,20,26 – D.lotus accessions.

ISSR 815

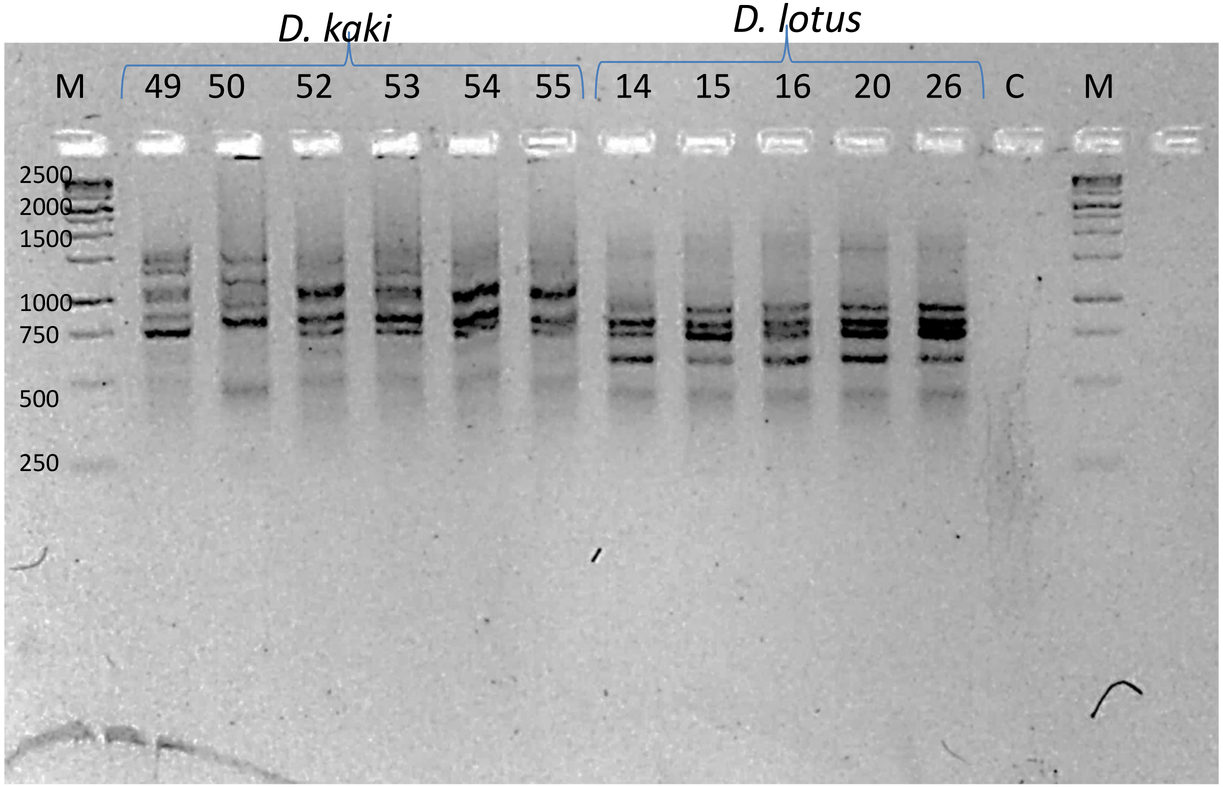

M – size marker, C – control; 49- D.kaki cv. ‘Tamopan’; 50- D.kaki cv. ‘Meader’; 52- D.kaki cv. ’Hachiya’, 53- D.kaki cv. ’Fuyu’, 54- D.kaki cv. ’Hyakume’, 55- D.kaki cv. ’Zenjimaru’; 14, 15,16,20,26 – D.lotus accessions.
