# Supplementary material for: Genetic Diversity in Diospyros Germplasm in the Western Caucasus Based on SSR and ISSR Polymorphism
Source: Biology (Basel). 2021 Apr 19;10(4):341. doi: 10.3390/biology10040341 (PMC8073590; doi:10.3390/biology10040341)
Supplement: Supplementary file 1 [file biology-10-00341-s001.zip › Supplementary table 3.docx]

**Supplementary table 3.** SSR primers used for the genetic analysis of the *Diospyros* germplasm collection

| SSR name | Primer sequence 5’-3’ | Repeat motif | Annealing T,^о^С | Amplicon size, bp |
| --- | --- | --- | --- | --- |
| ssrdk01 | F: CGGCATGAAGGAATAAGGAA  R: GCTCACATTCCAACCAATCA | (AG)19 | 60 | 155–184 |
| ssrdk02 | F: TTAATTTGGACACAAGTTCT  R: TCTCTTCAAGTCTTCTATCCT | (GA)17 | 50 | 196–224 |
| ssrdk03 | F: GGCTCTCGGTCAAATAGTAG  R: GGAGGTTAGAAATCCAGCTA | (AG)16 | 59 | 158–198 |
| ssrdk04 | F: CATTTGAAAGCAGTCGTCCA  R: GCGCCAAATCATTGCTATCT | (GA)17 | 60 | 336–365 |
| ssrdk06 | F: CGGCATGAAGGAATAAGGAA  R: GCTCACATTCCAACCAATCA | (AG)19 | 60 | 158–187 |
| ssrdk09 | F: ATGCCTCAAGCCTGTCATTT  R: GACATCCCTGTCATTTGAGGA | (AG)13 | 59 | 137–190 |
| ssrdk10 | F: CGACACTGATGGTTGATAAG  R: CAGCTTCACCTCCTAGAGAC | (GA)15 | 59 | 193–224 |
| ssrdk14 | F: GTGAAGGAACCCCATAGAA  R: CCATCATCAGGTAGGAGAGA | (AG)16 | 55 | 155–178 |
| ssrdk15 | F: AGAGAACAGAGAGGGAATAG  R: TTGGGATTAGTTGATTGTAG | (GA)9 | 59 | 235–254 |
| ssrdk16 | F: ACTACAACGGCGGTGAGAAC  R: GTCCTTCACTTCCCGCATT | (GA)12 | 59 | 134–173 |
| ssrdk17 | F: GGTGTTGGGATATTAATGCT  R: CTGCAGATTATAGGCACAAA | (GA)19 | 59 | 138–168 |
| ssrdk25 | F: GGGGTAATATGAATTGAATC  R: CTCAGAGAGGAGAAGAAATAG | (CT)15 | 50 | 229–283 |
| ssrdk26 | F: GGGAAATTAAGAGGGAAGAA  R: AGGAACTGGATCAGCATAAA | (GA)15 | 55 | 152–202 |
| ssrdk28 | F: CGAGCAAGTAGATGTTTATT  R: TCATGATGATTAAAGAGGAC | (GA)16 | 50 | 176–199 |
| ssrdk29 | F: ATCATGAGATCAGAGCCGTC  R: CACGTTAACGTTACGGAACA | (CCTTT)8 | 57 | 115–150 |
| ssrdk30 | F: TGGTGATCGTGGTAGTGGTT  R: GGCCTAATCTCTGTCCATCC | (TG)9(AG)17 | 59 | 137–275 |
| ssrdk32 | F: TAGAGCGGGAAAGATCGAGA  R: TACTTGGCGAGCAGTTAGCA | (GA)8 | 58 | 147–201 |
| ssrdk36 | F: GGGAAGAACAAAGAGAACTG  R: ACGAAGTTGTAATCCTGAGC | (GA)16 | 54 | 226–259 |
| ssrdk37 | F: CAAAATGAAGCCCATAAGAC  R: GTGAAAGTGTGGTTGGATTT | (CT)10 | 59 | 154–211 |
