# Supplementary material for: Genetic Diversity in Diospyros Germplasm in the Western Caucasus Based on SSR and ISSR Polymorphism
Source: Biology (Basel). 2021 Apr 19;10(4):341. doi: 10.3390/biology10040341 (PMC8073590; doi:10.3390/biology10040341)
Supplement: Supplementary file 1 [file biology-10-00341-s001.zip › Supplementary table 4.docx]

**Supplementary table 4.** ISSR primers used for the genetic analysis of the *Diospyros* germplasm collection

| ISSR name | Primer sequence 5’-3’ | Annealing T,^о^С |
| --- | --- | --- |
| ISSR810 | GAGAGAGAGAGAGAGAT | 53 |
| ISSR813 | CTCTCTCTCTCTCTCTT | 53 |
| **ISSR815** | **CTCTCTCTCTCTCTCTG** | 53 |
| ISSR851 | TATTATTATTATTAT | 53 |
| ISSR873 | CTTCACTTCACTTCA | 53 |
| **ISSR880** | **GGAGAGGAGAGGAGA** | 53 |
| **ISSR13** | **ACACACACACACACACC** | 53 |
| ISSR14 | TGTGTGTGTGTGTGTGG | 53 |
| **ISSR15** | **TCTCTCTCTCTCTCTCC** | 53 |
| **ISSR814.1** | **CTCTCTCTCTCTCTCTTG** | 53 |
